# Supplementary material for: Attenuated Total Reflectance-Fourier Transform Infrared (ATR-FTIR) Spectroscopy Discriminates the Elderly with a Low and High Percentage of Pathogenic CD4+ T Cells
Source: Cells. 2022 Jan 28;11(3):458. doi: 10.3390/cells11030458 (PMC8834052; doi:10.3390/cells11030458)
Supplement: Supplementary file 1 [file cells-11-00458-s001.zip › Suplementary Materials.pdf]

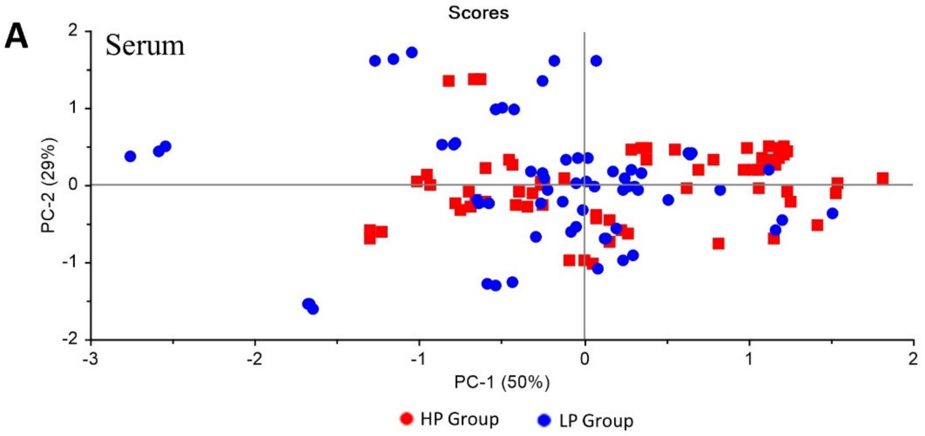


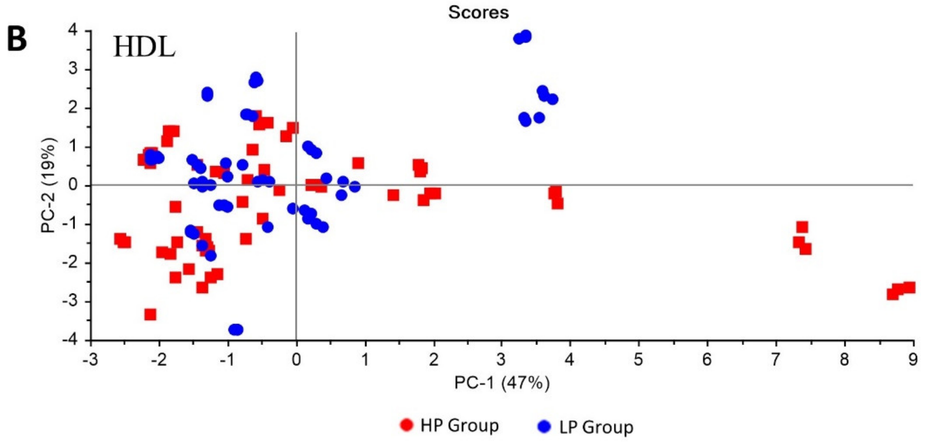


**Figure S1.** The representatives of PCA results from serum and HDL samples. PCA analysis was generated using the 1700-1500 $cm^{-1}$ FTIR spectral range. PCA score plots of serum (A) and HDL (B). The blue and red represent the LP and HP groups, respectively. Score plots of serum and HDL exhibited no trend of discrimination for the LP and HP groups. HP – the elderly with a high percentage of pathogenic CD4+ T cells; LP - the elderly with a low percentage of pathogenic CD4+ T cells.

**Table S1.** Confusion matrix of classification model built by PLS-DA using exosome spectra in the 1700-1500 $cm^{-1}$ region

| Predictive Model | Flowcytometric Analysis | |
|---|---|---|
| | **HP** | **LP** |
| **HP** | 11 | 9 |
| **LP** | 5 | 14 |

Abbreviations: HP – the elderly with a high percentage of pathogenic CD4+ T cells; LP - the elderly with a low percentage of pathogenic CD4+ T cells.

**Table S2.** Performance of classification model built by PLS-DA using exosome spectra in the 1700-1500 $cm^{-1}$ region

| Sample | Region ($cm^{-1}$) | Algorithm | Performance | | | | |
|---|---|---|---|---|---|---|---|
| | | | **Acc (%)** | **Sens (%)** | **Spec (%)** | **PPV (%)** | **NPV (%)** |
| Exosome | 1700-1500 | PLS-DA | 64 | 69 | 61 | 55 | 74 |

Abbreviations: Acc - accuracy; Sens – sensitivity; Spec – specificity; PPV – positive predictive value; NPV – negative predictive value; PLS-DA – Partial Least Square Discriminant Analysis.
