# Supplementary figures and images for: Attenuated Total Reflectance-Fourier Transform Infrared (ATR-FTIR) Spectroscopy Discriminates the Elderly with a Low and High Percentage of Pathogenic CD4+ T Cells
Source: Cells. 2022 Jan 28;11(3):458. doi: 10.3390/cells11030458 (PMC8834052; doi:10.3390/cells11030458)

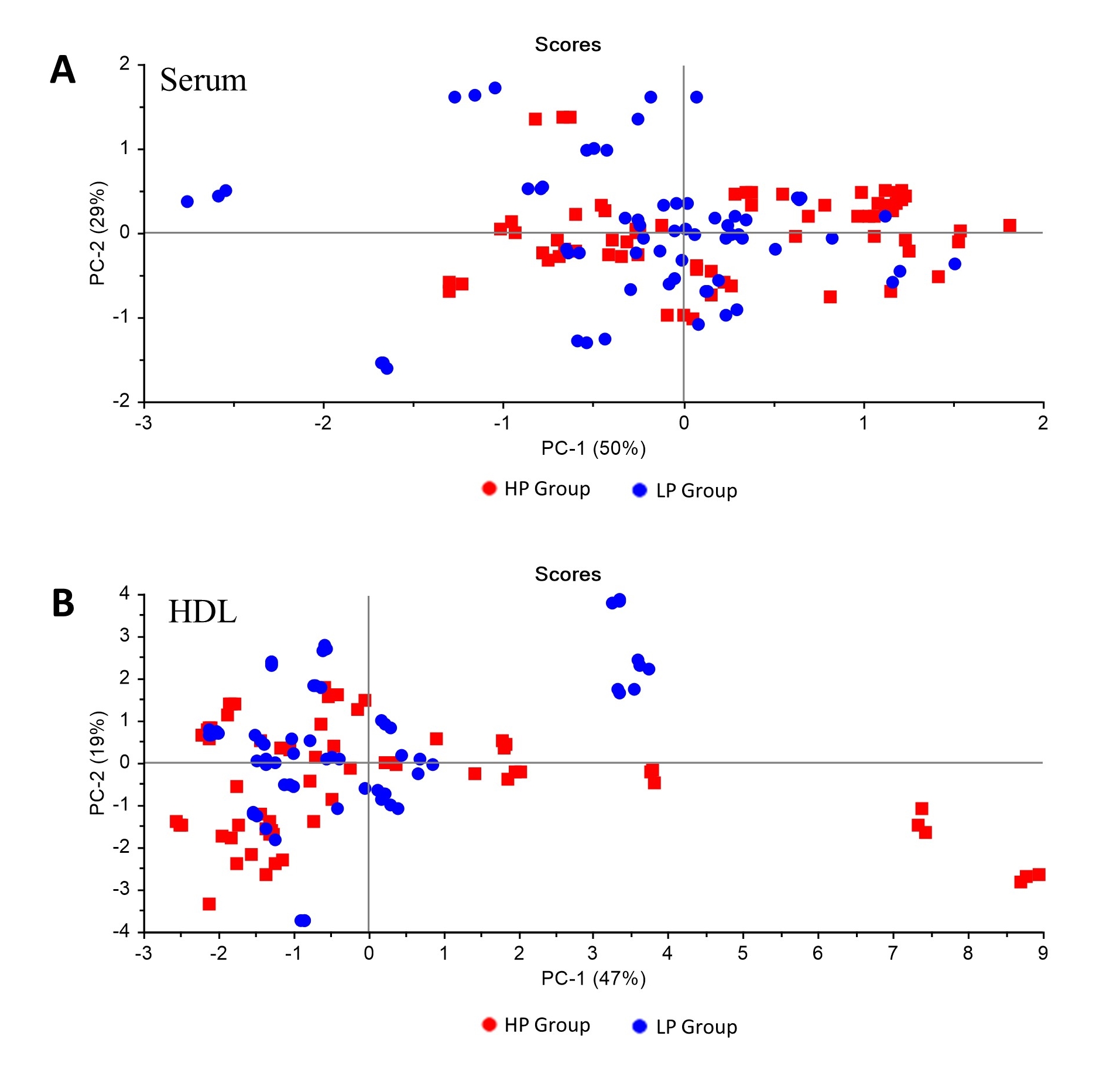

Supplement: Supplementary file 1 [file cells-11-00458-s001.zip › Suplementary Figure 1 - 17.11.21.jpg]
